# Discontinuous properties of current-induced magnetic domain wall depinning

**DOI:** 10.1038/srep03080

**Published:** 2013-10-30

**Authors:** X. F. Hu, J. Wu, D. X. Niu, L. Chen, S. A. Morton, A. Scholl, Z. C. Huang, Y. Zhai, W. Zhang, I. Will, Y. B. Xu, R. Zhang, G. van der Laan

**Affiliations:** 1 Spintronics and Nanodevice Laboratory, Department of Electronics, University of York, York YO10 5DD, UK; 2 Nanjing-York International Center of Spintronics, School of Electronics Science and Engineering, Nanjing University, Nanjing 210093, China; 3 Department of Physics, University of York, York YO10 5DD, UK; 4 Department of Electronics, University of Leeds, Leeds, LS2 9JT, UK; 5 Lawrence Berkeley National Laboratory, Berkeley, California 94720, USA; 6 Department of Physics, Southeast University, Nanjing 211189, China; 7 Diamond Light Source, Magnetic Spectroscopy Group, Chilton, Didcot OX11 0DE, UK; 8 Current address: Department of Physics, Southeast University, Nanjing, 211189, China.

## Abstract

The current-induced motion of magnetic domain walls (DWs) confined to nanostructures is of great interest for fundamental studies as well as for technological applications in spintronic devices. Here, we present magnetic images showing the depinning properties of pulse-current-driven domain walls in well-shaped Permalloy nanowires obtained using photoemission electron microscopy combined with x-ray magnetic circular dichroism. In the vicinity of the threshold current density (*J*_th_ = 4.2 × 10^11^ A.m^−2^) for the DW motion, discontinuous DW depinning and motion have been observed as a sequence of “Barkhausen jumps”. A one-dimensional analytical model with a piecewise parabolic pinning potential has been introduced to reproduce the DW hopping between two nearest neighbour sites, which reveals the dynamical nature of the current-driven DW motion in the depinning regime.

Ever since Berger^1^,^2^ and Slonczewski^3^ predicted spin angular momentum transfer of conduction electrons moving across the local magnetization due to mutual exchange coupling, the manipulation of domain-walls (DWs) by spin-transfer torque exerted from spin-polarized currents has attracted great interest in fundamental theoretical studies^4^,^5^,^6^ and promising potential applications, such as high density magnetic storage^7^ and logic devices^8^. To realize these devices based on current-induced DW motion (CIDWM), recent studies have naturally been focussed on operation speed and dissipation power of devices determined by DW velocity and operation current, respectively. Therefore, a high DW velocity and a low threshold current density (*J*_th_) are highly desirable for device applications.

Most studies of soft-ferromagnetic single-layer Ni_81_Fe_19_ (Py) nanowires with biaxial magnetic anisotropy (in-plane) and high Curie temperature suggest that the velocity of DW motion ranges from several m.s^−1^ 9,10 to ~100 m.s^−1^ 11 with current densities in the order of 10^12^ A.m^−2^, whilst Pt/Co/AlOx wires with perpendicular magnetic anisotropy even give a higher velocity of DW motion (nearly 130 m.s^−1^)^12^ under a similar current density *J* = 3.5 × 10^12^ A.m^−2^ and a maximum velocity of 400 m.s^−1^. Recent experiments of spin-valve devices report that the DW velocity can exceed 600 m.s^−1^ under current densities in the order of 10^12^ A.m^−2^ 13. However, the DW motion velocity at high current densities is limited by the so-called Walker breakdown^14^,^15^, where the internal DW structure periodically transforms between transverse and vortex walls. In addition, since spin-polarized currents can deform or change the spin configuration of DWs, the velocity depends on DW type as well as on pulse shape^16^,^17^. On the other hand, when the pulse current density is comparable to *J*_th_ (relative low current density) the DW generally needs to experience a depinning and motion progress before it is pinned. It has been established that the dynamical behavior of DWs in the depinning and pinning evolution becomes essential^18^,^19^,^20^, not only on the threshold current density *J*_th_, but also on the DW velocity in electrical measurements. In particular, magnetic imaging techniques, such as magnetic transmission x-ray microscopy, have provided a more direct understanding of the relationship between depinning and structure of the DW, under external magnetic fields^21^ as well as current pulses^22^. However, there are rare reports of the detailed depinning behaviour of a single DW in the vicinity of the threshold current density combined with an effective potential calculation under ns current pulses using direct magnetic imaging techniques.

Here, we report experimental results of a single DW depinning process under ns current pulses with amplitudes in the vicinity of the threshold current density, *J*_th_, in well-shaped Py using photoemission electron microscopy (PEEM) combined with x-ray magnetic circular dichroism (XMCD), also known as XPEEM. We will show that direct observation of DW depinning based on the magnetic imaging technique XPEEM gives different DW configurations corresponding to different depinning situations, which are complementary to transport measurements. In order to explain the overall “discontinuous” motion (Barkhausen jumps) of DWs we first simplify a random pinning potential of the real nanowire to a form of piecewise parabolic potentials within two pinning sites and then quantitatively depict pictures of DW hopping and depinning between both pinning sites, resulting from defects and roughness. Then based on detailed calculations we discuss the depinning boundary by taking into account the non-adiabatic term and the pulse width.

## Results

**Figure 1:**
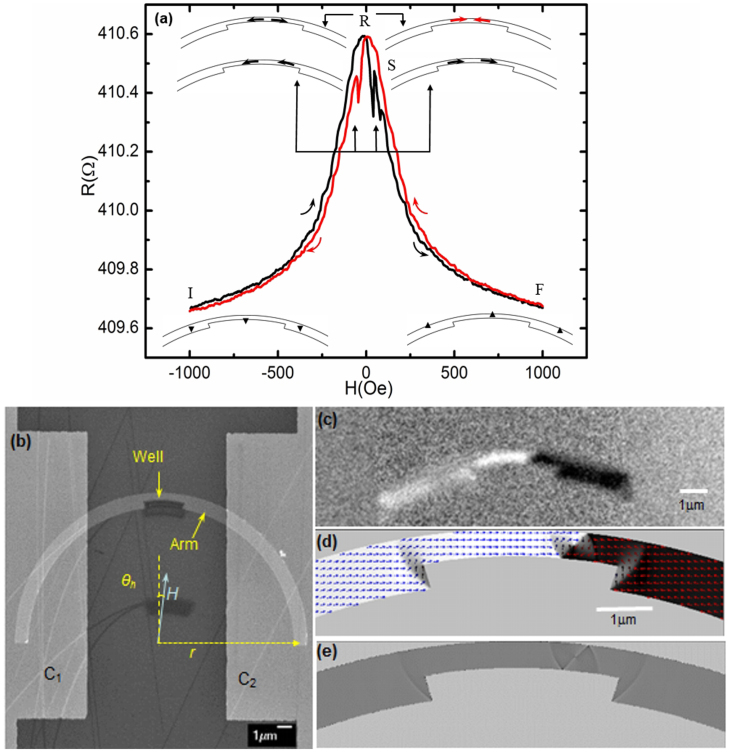
Nanowire characterization. (a) AMR at room temperature measured with transverse MR. The up-sweep (down-sweep) is plotted in black (red). Also shown are the schemes for the different domain configurations corresponding to different magnetization states during the whole transverse MR measurement cycle. (b) Scanning electron micrograph of the well-shaped Py nanowire with contacts C_1_ and C_2_, where *θ_h_* is the angle between *H* and the vertical direction and *r* is the radius of the overall pattern of the nanowire. (c) XPEEM image of the initial vortex DW (VW) which is located in the well-shaped part of the wire at a position defined by the angle *θ_h_* ≈ 4°. (d) Micromagnetic simulation of the initial VW state using OOMMF. (e) The image of 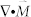.

Transverse magnetoresistance (MR) measurements of the nanowires were carried out at low direct current of 300 μA. As shown in Fig. 1a, the plot represents primarily the anisotropic magnetoresistance (AMR) properties^23^,^24^. The lowest resistance of DW corresponds to the saturation magnetization states I and F, because the current is mostly perpendicular to the magnetization. The highest resistance is reached at the remnant state R, where the magnetic field is zero, corresponding to the onion state with either tail-to-tail or head-to-head DW depending on the magnetic history. When the magnetic field gradually increases from zero to 50 Oe or decreases to −50 Oe, the resistance shows a sharp increase indicating that at this field the DW depins corresponding to the magnetic globe-vortex state S^25^,^26^.

Therefore, the static AMR measurements not only help us to detect a DW but also allow us to discriminate between different DW spin structures^27^, although it cannot disclose the position and displacement of the DW, except in the case of time-resolved AMR measurements^28^ or magnetic imaging techniques.

**Figure 2:**
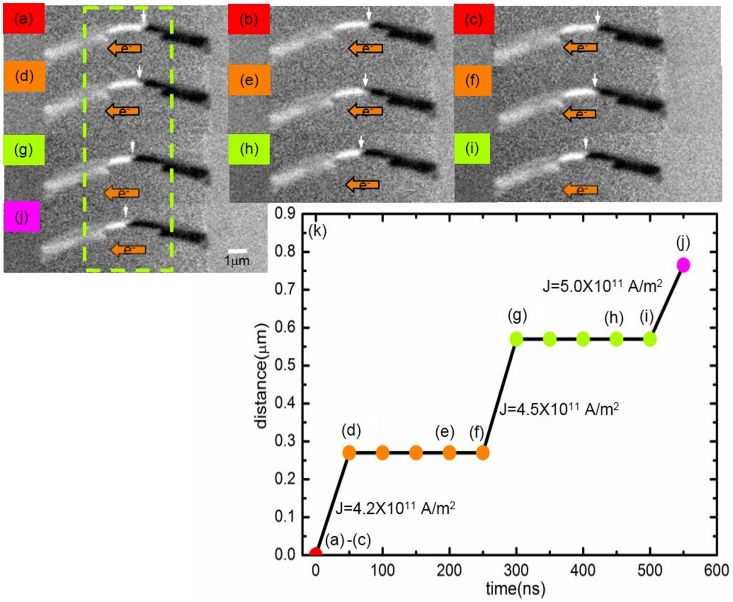
XPEEM images of a single DW in a nanowire. (a)–(j) Sequence of images captured after separate 50 ns current pulses with stepwise increasing current densities, *J*. The white arrow heads are a guide to the eye to mark the DW position. The dashed green box in the first column of images shows all the DW depinning. (k) Plot of the DW motion distance against time. The discontinuous properties of the DW motion are revealed by the time evolution under pulses of different current densities. The size of circles denotes the error bar during the measurements. The images c, f and i in the plot show the last state prior to each depinning. The current densities at which the DW depins are indicated.

After AMR measurements, images of the magnetic domain structures were captured using XPEEM^29^ in zero field and at room temperature. Figure 2 shows the XPEEM images of a single DW depinning process under a series of current pulses of 50 ns with stepwise increasing amplitudes (of the current density). For the initial DW, shown in Fig. 2a (also shown in Fig. 1c), current pulses of 50 ns are injected with amplitudes starting at 3.5 × 10^10^ A.m^−2^ gradually increasing to 5.0 × 10^11^ A.m^−2^. For current densities below 4.2 × 10^11^ A.m^−2^ there is no depinning of the DW, as seen in Fig. 2a–c, whereas DW depinning occurs for the first time at 4.2 × 10^11^ A.m^−2^. Figure 2d shows a forward DW motion of ~270 nm, while the four successive measurements with pulses of the same amplitude do not give depinning, as seen in the next two images e and f. Increasing in amplitude to 4.5 × 10^11^ A.m^−2^ gives depinning with forward DW motion of ~300 nm, as seen in Fig. 2g. However when the same pulse is injected again four times, the DW does not depin, as shown in images h and i. After increasing the current density to 5.0 × 10^11^ A.m^−2^ there is again a clear depinning with 250 nm motion, as shown in image j. At those stages, where the DW depins, the average DW velocity defined as the displacement divided by current pulse length is ~5 m.s^−1^, with the DW moving in the electron flow direction. These velocities are much smaller than those observed in experiments where the DW is in the flow regime at a higher current density^11^ or is triggered by a magnetic field pulse^30^. Figure 2k shows the overall progress of the DW depinning, which clearly reveals the discontinuous properties. This is also known as a sequence of Barkhausen jumps^31^,^22^, which is a normal phenomenon in the presence of field-driven magnetization reversal. Since the disorder correlated random pinning potential in the nanowires is responsible for the jerky motion^32^ of field-driven Barkhausen effect, we propose that a similar mechanism is present in the case of current-driven DW depinning, in which there are pinning sites resulting from defects corresponding to a series of random potentials which play an essential role.

### Micromagnetic simulation and theoretical investigations

In first instance, we do not include the thermal perturbation induced depinning, which will be discussed afterwards. In order to obtain deeper understanding of the discontinuous depinning we implemented a modified version of OOMMF software^33^, where the Landau-Lifshitz-Gilbert (LLG) equation is extended by adding the adiabatic and nonadiabatic spin transfer torque (STT) terms^15^,^34^ as 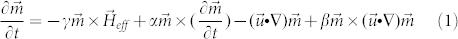
where 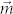, *γ*, *α* and *β* are the unit vector along the local magnetization, the gyromagnetic constant, the Gilbert damping factor and the dimensionless non-adiabatic spin-transfer parameter describing the strength, respectively. 
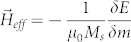
 is an effective field which includes the external field, anisotropy field, magnetostatic field and exchange field, 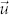 is a vector pointing along the electron direction with absolute value *u* = *j_e_Pgμ_B_*/2*eM_s_*, where *M_s_* the saturation magnetization, *j_e_* the current density, *P* the spin-polarization of the current, *g* the electron *g*-factor, *μ_B_* the Bohr magneton and *e* the electron charge.

**Figure 3:**
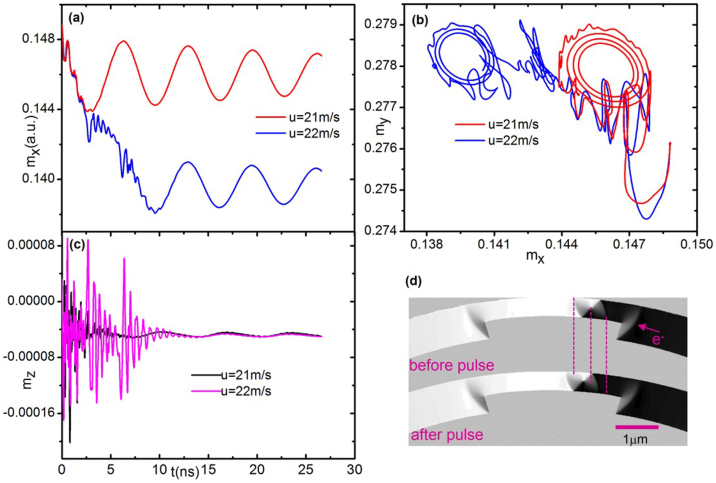
Simulation of a spin-polarised current pulse with current density 1.0 × 10^12^ A.m^−2^ on a single VW in a well-shaped Py wire with similar structure as Fig. 1b. (a) *m_x_* (*t*) for different current pulse amplitudes, where *m_x_* is the *x* component of the magnetization of the whole nanowire (along its length) rather than the localised magnetization of the DW. (b) The trajectories of *m_y_* with respect to *m_x_* in the wire. (c) *m_z_* (*t*) of the whole wire. (d) Simulated VW motion under the threshold current density. The DW has moved forward by ~250 nm.

Figure 3 shows a simulation of a single VW under a current pulse below the threshold current density 1.0 × 10^12^ A.m^−2^ (*u* = 22 m.s^−1^ and *P* = 0.3), which is nearly twice as large as the experimental results.

**Figure 4:**
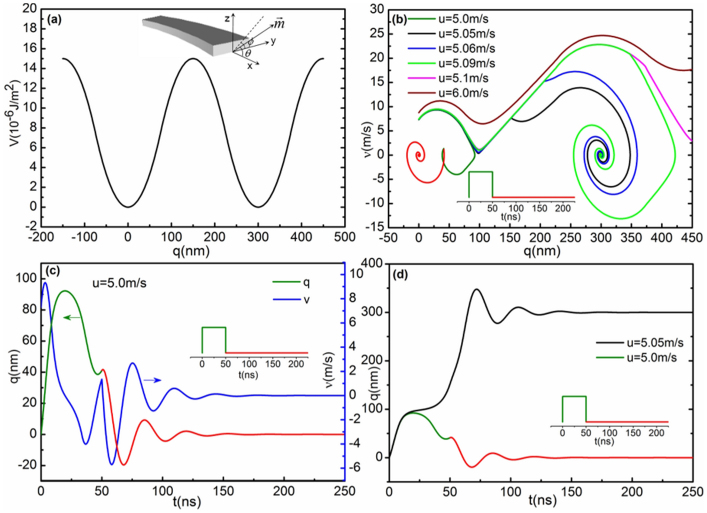
1D calculation of DW pinning and depinning. (a) Pinning potential *V_pin_* (*q*) with *d* = 75 nm, which means that the distance between two pinning sites is 300 nm.*V*_0_ = 0.1 × 10^3^ J.m^−3^.The inset shows schematically the magnetic nanowire with corresponding coordinate system. (b) Trajectories of the DW in phase space (*ν* vs *q*) with the rigid 1DW under different current pulses of fixed length [different *u* values and *t_p_* = 50 ns (inset corresponds to *u* = 5.0 m.s^−1^)]. (c) Plots of *q*(*t*) and *ν*(*t*) for the pinning case below threshold current density. (d) Plot of *q*(*t*) for DW below and above threshold current density. The calculations have been done by using *α* = 0.01,*β* = 0.04, Δ = 14 nm and *H_k_* = 500 Oe.

Writing the DW position as *q*(*t*), the *x* component of the magnetization of the whole nanowire can approximately be expressed as *m_x_* = (2*q*/*L*) cos[(*L*/2 − *q*)/*r*], (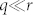) with length *L* of the wire and a radius *r* of 10 μm as shown in Fig. 1b. Therefore, the simulated variable *m_x_* can be used to certain extent to show the DW position. Since the DW moves forward, the negative *x* direction, *m_x_*, decreases during DW motion. When the current density of the pulse is below its threshold value the DW will oscillate within the pinning site, whilst the DW will jump to a new pinning site when the current density reaches the threshold value as shown in Fig. 3a. To save in computing time, we have only simulated the case for a pulse width of 27 ns, after which there is no need to push the DW. The trajectories of *m_y_* with respect to *m_x_* in the wire also show the pinning and hopping of the DW, although there is much chaos at the initial stage, as seen in Fig. 3b. Additionally, the depinning properties shown in the trajectories of *m_y_* with respect to *m_x_* can also be presented by trajectories of the DW in phase space, as shown below in Fig. 4b. In addition, the amplitude of *m_z_* oscillates but is very small as seen in Fig. 3c. This means that when the DW pins and depins at lower current densities the DW's plane tilts slightly away from in-plane in the wire. During the above regimes the linearized condition about *ϕ* (mentioned below) is satisfied. Furthermore, the extent of tilting will become stronger under larger current densities. When the current approaches the Walker breakdown, *ϕ* becomes very large and the above condition will default. The simulation results clearly embody the hopping properties of the DW depinning progress, in agreement with the experimental results, except that the threshold current density in the simulation is twice as large as in the experiments. Finally, Fig. 3d shows that the DW moves forward by ~250 nm, which is close to experimental values.

Next, we will focus on the analytical insight in the DW dynamics depinning to reproduce the pictures of the hopping properties using a one-dimensional (1D) model^35^ based on Eq. (1). Since the radius of the overall pattern of curved well-shaped nanowires is nearly 10 times larger than the DW motion displacement, the 1D model can be used approximately. Taking the DW motion direction as the coordinate axis *x* and denoting *θ*(*x*,*t*) as the magnetization polar angle with respect to the strip axis, i.e., *x* and *ϕ*(*x*,*t*) as the azimuthal angle, which describes the orientation of the magnetization projection onto the *y-z* plane, shown in the inset of Fig. 4a, 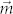 can be written as 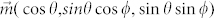. Eq. (1) in the 1D framework is 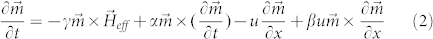
where 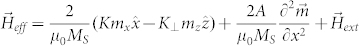, *K* is the magnetic anisotropy energy density along the easy *x*-axis in-plane, while 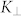 is the magnetic anisotropy energy density along the hard *z*-axis out-of-plane and *A* is the exchange strength coefficient. In order to include the effect of DW trapping by defects, 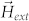 contains apart from the external magnet fields also the effective pinning field 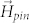. In the case of current induced depinning there is no external magnetic field but only a pinning field, so that 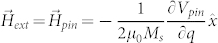
36, where *q* is the DW's central position on the *x* axis. Substituting 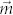 and 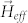 into Eq. (2) and combining with the wall profile, which can be predicted by the Walker trial function^14^,^15^,^37^

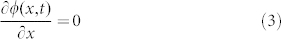

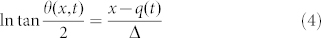
results in two equations with two main collective variables of DW motion^19^,^20^

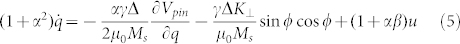

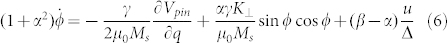
with effective DW width, 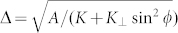. Introducing the anisotropy field 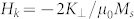 and considering that *ϕ* is very small during DW depinning when the current is below the Walker breakdown, Eq. (5) and (6) can be linearized using sin *ϕ* ≈ *ϕ* and become second-order ODEs in *q* and *ϕ*, respectively.

To obtain an analytical solution and to make pinning potentials close to the real situation with physical rationality, we introduce a piecewise parabolic function represented as 
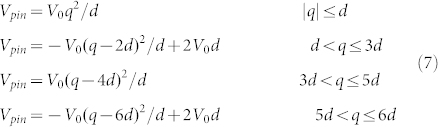
where *V*_0_ is the depth of the potential (in units J.m^−3^), *d* is the spatial extension and 4*d* equals the distance between two pinning sites shown in Fig. 4a. This kind of piecewise potential can not only be used to represent the depinning progress with the same function of the single parabolic function^19^,^20^ but is also very similar to the potential of an exponential function^36^,^38^ in second-order approximation, which can describe the hopping properties. However, it is simpler and can provide analytical solutions while to certain extent retaining the intrinsic defects properties. In our case, where *d* > 5Δ and *H_k_M_s_μ*_0_ > 10*V*_0_, additionally considering the initial conditions that *q_i_* = 0 and 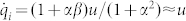, so we can approximately obtain analytical solutions of the DW motion in the different potential regions for both cases where a current pulse is switched on or off. Furthermore, we can obtain two key physical quantities, namely the characteristic time, *τ* = 2*M_s_μ*_0_*d*/[*αγ*(Δ*V*_0_ + *M_s_μ*_0_*dH_k_*)], and the oscillation frequency, *ω* = γ(Δ*V*_0_*H_k_*/*M_s_μ*_0_*d*)^1/2^. Dynamical pictures of DW pinning, depinning and hopping between two local pinning sites due to defects or roughness in the nanowire are shown in Fig. 4b–d.

**Figure 5:**
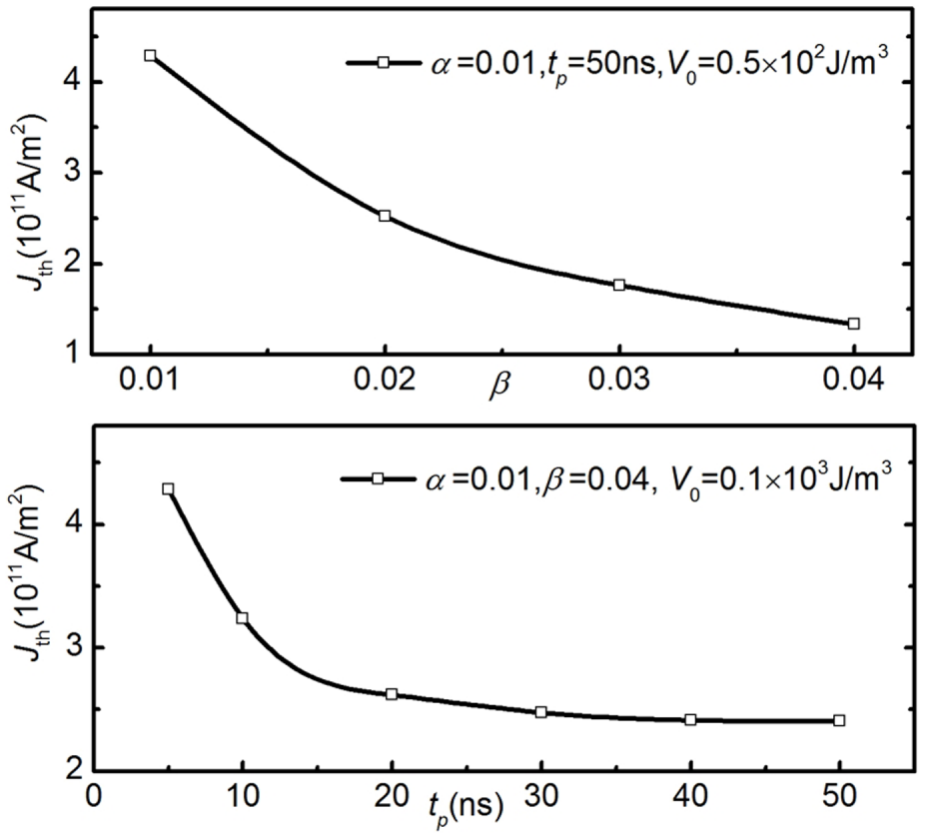
Domain wall depinning boundary as a function of (a) the nonadiabatic torque term, *β* and (b) the current pulse length, *t_p_*.

Figure 4b illustrates the trajectories of the DW in phase space for different current densities of 50 ns. Assuming *d* = 75 nm in nearest-neighbour hopping, although this depends not only on the physical size of the defects but also on DW profile^19^,^20^, obviously the threshold value *u*_th_ corresponding to the threshold current density satisfies *u*_th_ = *u*_th_ (*α*, *β*, *t_p_*, Δ, *H_k_*, *V*_0_). Here, we take *α* = 0.01^19^,^20^,^39^, *β* = 0.04^15^,^19^,^20^,^40^, while Δ = 14 nm and *H_k_* = 500 Oe are estimated from micromagnetic simulations for a VW. *V*_0_ is obtained by matching the *u*_th_ value to the experimental value. For permalloy, *P* ranges between 0.3–0.45^41^ and if we choose *P* = 0.3 then *J*_th_ ≈ 2.33 × 10^11^ J.m^−2^, which is almost half the experimental value. For larger current densities than this, the DW hops to the nearest neighbour pinning site, while for smaller values the DW returns to its initial position. Figure 4b also shows that when *u* = 5.1 m.s^−1^, i.e., *J* = 2.4 × 10^11^ J.m^−2^, the DW will exceed the nearest-neighbour hopping and may stay at the next-nearest-neighbour pinning sites. With the increasing of the pulse current density the DW motion gradually enters the flow regime and finally reaches the Walker breakdown point. Figure 4c shows *q*(*t*) and *ν*(*t*) with the pinning case below threshold current density, whereas Fig. 4d describes *q*(*t*) for DW pinning and hopping, respectively. Since the values of *α* and *β* and their relations are still under debate, their present values give only one of the possible solutions. If we increase *β* from 0.01 to 0.04 with *V*_0_ = 0.5 × 10^2^ J.m^−3^ then *J*_th_ decreases from 4.29 × 10^11^ to 1.33 × 10^11^ J.m^−2^, as shown in Fig. 5a. This means that in order to reduce the threshold current density, the search for materials with higher non-adiabatic torque provides an opportunity. However, a non-adiabatic torque is influenced by other effects, such as the Oersted field (created by the current itself), the spin-orbit coupling and even magnetization gradients. Therefore, the dependence of *β* on the materials properties calls for further experimental and theoretical investigations. In order to investigate the dependence of *J*_th_ on the current pulse length we keep the parameters the same as in Fig. 4b. The result for *t_p_* = 5–50 ns is shown in Fig. 5b. With increasing pulse length, *J*_th_ gradually decreases, but will approach 2.33 × 10^11^ J.m^−2^, below which the depinning is apparently frozen. The calculated result is essentially in agreement with experiments^40^, providing a so-called depinning time. Current pulse lengths larger than this do not result in a lower *J*_th_, except for potential thermal effects. Defining this type of depinning time by a characteristic time, the present case amounts to ~22.7 ns. From Fig. 5b we can also see that *J*_th_ still changes slightly when the pulse length is larger than 22.7 ns. In fact, from the perspective of the competition between the driving force *F*_j_ of the current pulses and the pinning force *F*_pin_(*q*) of the pinning potential, when *F*_j_ is less than a certain value of *F*_pin_(*q*) the DW will oscillate around a certain equilibrium point irrespective of pulse length. When the pulse is switched off, the DW returns to the initial position. Figure 5 also shows that different potentials give different threshold current densities. For 50 ns pulse length, *V*_0_ = 0.1 × 10^3^ and 0.5 × 10^2^ J.m^−3^ give *J*_th_ = 2.33 × 10^11^ and 1.33 × 10^11^ J.m^−2^, respectively. Additionally, from viewpoint of the pinning frequency *ω*, we obtain that the single-DW mass is *m* = 2*M_s_μ*_0_*S*/Δ*H_k_γ*^2^ = 0.89 × 10^−23^ *kg* (*S* is the cross-section area), which is comparable to the value obtained in resonance motion of the DW induced by an oscillating current^42^ as well as with the theory^43^.

Based on the above theoretical analysis, we return to the discontinuous depinning in Fig. 2. Due to the dependence of the threshold current density on *β* and *V*_0_, when DW arrives at different positions in the nanowire, it will encounter different *V* values resulting from different defects, roughness of the rim and even DW structure. In addition, from Fig. 2 we can see that the DW configuration changes during its motion, which may also result in different *β* values corresponding to different DW structures. In other words, the threshold current density remains the same within experimental error for the same position and DW structure. Although the above analytical solution with an effective potential does not take into account the change in DW configuration, DW width and spin-waves excitations, it still shows the essential properties of the DW depinning in the vicinity of the threshold current density.

## Discussion

We have assumed, in the above analysis, that no thermal activation effects were present. With a current density close to 10^11^ A.m^−2^, if we would assume that only the Py nanowire absorbs the heating power, the sample heating would rise at the enormous rate of nearly 30 K.ns^−1^, increasing the temperature of the nanowire close to its Curie point. However, the substrate plays an essential role in the heat dissipation. Regarding our sample, considering the natural oxidation of the Si substrate, the actual substrate consists of Si (500 μm)/SiO_2_ (~2 nm). Both from theoretical^44^,^45^,^46^ and experimental^40^ perspective, the temperature rise of the wire is Δ*T* ≈ 2–3 K. In addition, our AMR results at zero Oe for different currents (from 100 μA to 1 mA) show that the change ratio in resistance due to thermal effects is ~0.17%. Thus when we choose *V*_0_ = 0.1 × 10^3^ J.m^−3^ from the above analytical results, the energy barrier is of the order of 10^−19^ J, which is two orders of magnitude larger than *k_B_T* at room temperature. Thus the probability of thermal activated depinning is very low. However, if the SiO_2_ layer would be much thicker, e.g., 100 nm, the situation becomes very different^46^,^47^. The Si substrate without the interlayer or with only a very thin interlayer will significantly suppress the Joule heating^48^.

Using the XPEEM technique combined with simulations and analytical model calculations we have investigated the detailed DW depinning process in Py nanowires. We have observed a current-driven “Barkhausen jumps” of the DW depinning and motion, a similar phenomenon to the classical magnetic field-driven Barkhausen effect. The discontinuous properties of the DW depinning can be explained by a nearest-neighbour hopping picture based on STT rather than a thermal activation effect. The depinning boundary with nonadiabatic term and the pulse length based on the analytical model using a simple piecewise parabolic function can qualitatively explain the experimental observations. Our observations confirm that if the configuration of the DW stays the same their depinning should be similar within the error bar. The so-called stochastic properties result from the “stochastic” deformation of the DW shown as a non-adiabatic term and potential. The DW configuration may change during its motion, which results in different *β* values corresponding to different DW structures. However, the threshold current density remains the same for the same position and DW structure, and the stochastic depinning and motion related to thermal activation have little effect, which is critically important to the applications of the next generation CIDWM based spin devices.

## Methods

### Samples

Curved and well-shaped Ni_81_Fe_19_ (Py) nanowires of 10 μm radius were fabricated using electron-beam lithography (EBL) and lift-off, while the well part of the wires was patterned using focused-ion beam (FIB). The arm of the wire is 950 nm wide and the well is 450 nm wide and ~3.5 μm long, as shown in Fig. 1b. The 20 nm Py layer was deposited by thermal evaporation on undoped Si(100) substrate, followed by 2 nm Au protection layer to prevent oxidation. The second level electrodes and top electrodes made from Au were fabricated by EBL and photolithography, respectively. The curved wires were designed to facilitate the creation of a single DW under external magnetic field. Furthermore, the well-shaped structure helps to confine the DW within a relative small area.

### XPEEM images

Magnetic images of the domain structures were captured with XPEEM^29^ in zero field and at room temperature using the PEEM III end-station on beamline 11.0.1.1 at the Advanced Light Source at Lawrence Berkeley National Laboratory, California. XPEEM images of the DW structure are acquired from the ratio of the x-ray absorption signals at the Ni *L*_3_ and *L*_2_ edges measured with right-circularly polarized x-rays. The magnetic contrast of the domains is proportional to the magnetization component along the x-ray helicity vector, so that domains aligned parallel and antiparallel to the right-circular polarization vector show up in white and black color, respectively, as shown in Fig. 1c. The DW boundaries can be detected with an accuracy of ~30 nm. Prior to applying the current pulses to the sample in the main chamber, an initial DW was created within the well part of the nanowire by using a magnetic field sweep from zero to 1000 Oe and then back to zero with the applied field perpendicular to the overall direction of the nanowire. The actual current of the injected pulses flowing through the nanowire was obtained from the voltage measured using the 50 Ω input impedance of an oscilloscope connected in series with the nanowire.

### Micromagnetic simulation

The object-oriented micromagnetic framework (OOMMF) simulation^49^ was used to realize one of the initial DW structures, which is a head-to-head vortex DW (VW) as in Fig. 1d–e. These are in accord with the results reported by other authors^50^,^51^. The typical parameters for Py were used with damping constant *α* = 0.01. The initial DW configuration depends not only on the nanowire geometry but also on the magnetic field angle, the magnetic history and the damping coefficients. A modified version of the OOMMF software^33^ was employed to simulate the hopping properties of CIDWM for two nearest neighbour pin sites. The material parameters of Py are the exchange constant of *A* = 1.3 × 10^−11^ J.m^−1^, saturation magnetization of *M_s_* = 800 kA.m^−1^, and damping parameter *α* = 0.01 and *β* = 0.04.

## Author Contributions

J.W. and Y.B.X. proposed this work; X.F.H., D.X.N., L.C., I.W. and Z.C.H. fabricated samples; J.W., S.A.M., A.S. and W.Z. did XPEEM measurements; X.F.H. did MR measurements and performed analysis of the data; Y.B.X., Y.Z., R.Z. and G.L. supported the experiments and the analysis of the data; X.F.H., G.L. and Y.B.X. wrote the manuscript.
